# “I have suffered something”: traumatic childbirth in 19th-century Britain

**DOI:** 10.1136/medhum-2023-012883

**Published:** 2025-03-26

**Authors:** Jessica Cox

**Affiliations:** 1 English, Brunel University London, Uxbridge, UK

**Keywords:** history, medical humanities, midwifery, obstetrics, pregnancy

## Abstract

In 1994, the American Psychiatric Association revised its definition of trauma in relation to post-traumatic stress disorder (PTSD), enabling the recognition of childbirth as a potentially traumatic event leading to the development of symptoms of PTSD. This article considers clinical definitions of postpartum PTSD in relation to 19th-century case histories of difficult childbirth, and posits that the circumstances of some of these births—particularly in the context of higher infant and maternal mortality—mean they were likely to have been experienced as highly traumatic events, which may have led to the onset of symptoms today associated with postpartum PTSD. While resisting problematic retrospective diagnoses of postpartum PTSD, the article highlights the presence of the now widely recognised risk factors for the disorder in the experiences of these women, and demonstrates that birth in 19th-century Britain had significant potential to be experienced as a traumatic event for mothers. In doing so, it seeks to contribute to a wider conversation around—and expand our understanding of—women’s (physical and emotional) experiences of childbirth at this time, as well as some of the medical practices commonly employed in the birthing room, and the ethical questions which emerge from some of these. The article begins by outlining the risk factors now associated with postpartum PTSD, before exploring these in relation to 19th-century birth narratives. It draws on medical case notes (primarily the case studies of Dr Robert Lee) and women’s own accounts of childbirth, as well as advice literature for women on the subject of childbirth. The discussion focuses in particular on three issues: women’s knowledge around childbirth and agency within the birthing room (including issues of consent); the use of interventions in childbirth; and infant loss. The final part of the article briefly considers 19th-century discourses around puerperal insanity, and notes an association between difficult deliveries and the onset of puerperal insanity in some cases.

## Introduction

 In February 1824, a woman named Mary Stephenson, aged 32 years, was admitted into the Newcastle Lying-in Hospital in northeast England in labour with her fourth child. Her first labour had taken place some 12 years previously, but the baby could not be safely delivered due to Mary’s malformed pelvis—possibly the result of rickets as a child. An embryotomy (surgical destruction of the fetus) had been performed to enable the extraction of the infant from the mother’s body, thus preserving her life. In July 1820, Mary had delivered a living child, a daughter, at 7 months. It is unclear if or for how long the child survived. Her third child was delivered, again by embryotomy, at the Newcastle Lying-in Hospital in November 1822. On this occasion, the procedure followed more than >4 days in labour, and a failed effort, lasting some 2 hours, to deliver the child using the Vectis (a single-blade instrument used to try and adjust the position of the baby’s head). The doctors attending her fourth labour initially again anticipated the destruction of the infant in order to ensure delivery and the survival of the mother, but this time the child (another daughter) was delivered alive at full term. Mary, however, fell ill shortly after the delivery with symptoms of puerperal fever and died 3 days later (see Medical Report Book for Newcastle Lying-in Hospital July 1821–March 1833).

Mary Stephenson’s experience of childbirth was deeply traumatic: she lost two of her four children in a particularly devastating way, underwent a premature labour, for which the survival chances for the child would have been slim, as well as protracted labours that were subject to lengthy instrumental interventions. If her experiences of childbirth were not entirely typical of those of most 19th-century mothers, neither were they particularly unusual during this period.

**Figure 1:**
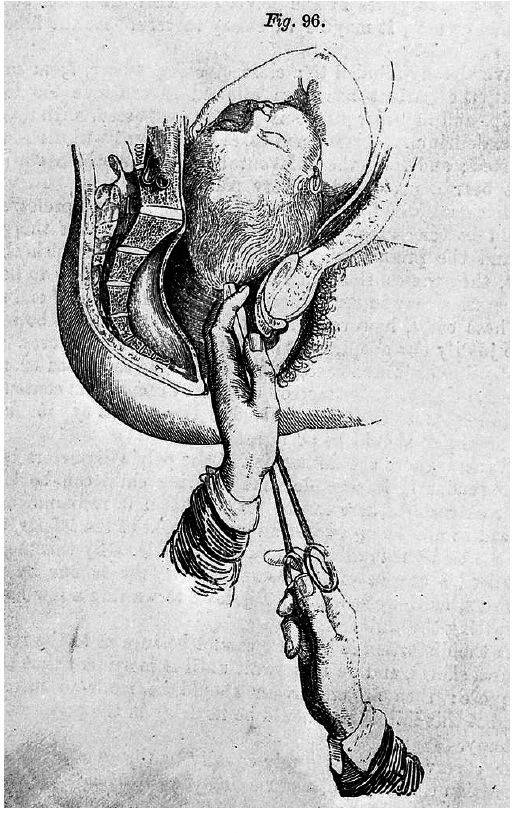
‘Use of the perforator’. Source: *On the Theory and Practice of Midwifery* (1855) by Fleetwood Churchill. Wellcome Collection.

**Figure 2:**
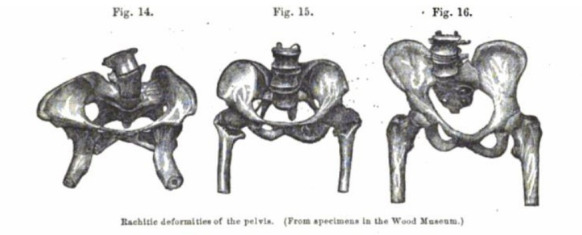
‘Rachitic deformities of the pelvis’. Source: Ashhurst 1881. *The International Encyclopedia of Surgery*, Volume I. New York: William Wood and Company, 267.

Over the course of the 19th century,^1^ thousands^2^ of destructive operations—craniotomies and embryotomies^3^ (see figure 1)—were performed in Britain on unborn children to ensure delivery and thus preserve the life of the mother at a time when caesarean sections carried a far greater mortality risk to mothers, and were thus rarely performed in Britain.^4^ Often, though not always, the infant would already have died before the operation was undertaken. Though the subject of intense ethical debates, there was nonetheless general agreement that “the life of the mother is by far of the greatest importance” (Parr 1819, 302). Rickets, a bone disease caused by lack of vitamin D leading in some cases to deformation of the pelvis and thus subsequent difficulties in delivering children (see figure 2), was widespread, particularly among the poorer classes (see Hardy 2003, 337–40), and a key factor in the performance of these operations.^5^ Mary Stephenson, like other mothers at this time, received little in the way of pain relief, and instrumental interventions could be particularly painful.^6^ Medical records also suggest that such interventions posed a significant risk of injury to the mother, in some cases resulting in devastating, long-term damage caused by lacerations inflicted by the instruments during delivery. In some cases, this resulted in permanent incontinence as a consequence to the bladder or perineum. Infection—often caused by a lack of basic hygiene practices by doctors, stemming from a limited understanding about the spread of germs—was also relatively common, painful and one of the leading causes of maternal mortality, which across the century averaged approximately 0.5%: while most women survived childbirth, the figure of one in every 200 births resulting in the death of the mother was far higher than today (see Loudon 1986).

Following her first labour, Mary would have had some awareness of the risks to her and her unborn child during subsequent labours and deliveries. It is possible her second child was deliberately delivered early to try and avoid these issues, as was relatively common practice,^7^ despite the poor survival rates for children born prematurely in the 19th century. Following the second embryotomy, doctors recommended she deliver any subsequent children prematurely, but she appears to have ignored their advice in the case of her fourth pregnancy. As a married woman, she would have had little control over whether or not to bear further children, despite the risks. Contraceptive options were limited (see McLaren 2022), and she had no legal right to refuse sexual relations with her husband.^8^ Each of her confinements must have been deeply traumatic—both physically and emotionally. Her medical records, however, while recording her physical health, details of her labours and information about the various procedures carried out, make no reference to her psychological or emotional state. In this respect, they are indicative of a wider trend in 19th-century medical discourses around childbirth. While some of the literature around puerperal insanity suggests a link between difficult births and the onset of this disorder, on the whole, there is little interest in women’s emotional and mental responses to traumatic births.^9^ In some respects, this is not surprising: postnatal post-traumatic stress disorder (PTSD) was not formally recognised until the late 20th century. Yet the risk factors now associated with this diagnosis were relatively common for women giving birth in 19th-century Britain, particularly protracted and/or difficult labours, instrumental interventions, risks to the life of both mother and child and a lack of control on the part of the patient.

This article examines some of the recognised risk factors associated with postpartum PTSD in relation to 19th-century case studies of difficult parturitions. The aim is not to attempt to retrospectively diagnose 19th-century mothers with postpartum PTSD, but rather to highlight the presence of the now widely recognised risk factors for the disorder in the experiences of these women, and to evidence that birth in 19th-century Britain had significant potential to be experienced as a traumatic event for mothers. The article begins by outlining these risk factors, before exploring them in relation to 19th-century birth narratives dating from the 1820s to the early 1900s. It draws on medical case notes (primarily the work of Dr Robert Lee,^10^ who detailed several hundred incidents of difficult partitions in his published work, which was aimed primarily at medical practitioners),^11^ women’s own accounts of childbirth (specifically those included in *Maternity: Letters From Working Women*—published in 1915 by the Women’s Cooperative Guild, and detailing women’s maternal experiences from the late 19th and early 20th centuries), as well as 19th-century advice literature for women on the subject of childbirth—a genre which burgeoned over the course of the period, as motherhood became increasingly marketed. The discussion focuses in particular on three issues: women’s knowledge of childbirth and agency within the birthing room (including issues of consent); the use of interventions in childbirth (including forceps delivery and destructive operations) and mothers’ experiences of infant loss. The final part of the article briefly considers 19th-century discourses around puerperal insanity, and notes an association between difficult deliveries and the onset of puerperal insanity in some cases, as well as a number of parallels in terms of the symptoms of puerperal insanity and those now recognised as indicative of postpartum PTSD. In exploring these issues, the article seeks to expand critical understandings of women’s experiences of childbirth in 19th-century Britain, as well as the medical practices associated with it. Contingent to the discussion are questions of power, hierarchies of (medical) knowledge, medical ethics, the relationship between medicine and the law and the emotional experiences of birthing women. These issues are touched on at various points, although a detailed exploration of them lies largely outside the scope of this article, the primary focus of which is to consider women’s potentially traumatic experiences of childbirth in 19th-century Britain.

## Risk factors for postpartum PTSD

In 1994, the American Psychiatric Association published the fourth edition of *The Diagnostic and Statistical Manual of Mental Disorders* (*DSM-IV*). This edition was significant for its revision of the definition of trauma as it pertains to events leading to the development of PTSD—a condition first formally recognised in *The DSM-III*, published in 1980. The entry on PTSD in *The DSM-III* notes that:

The essential feature of this disorder is the development of characteristic symptoms following a psychologically distressing event that is *outside the range of usual human experience* (ie, outside the range of such common experience as simple bereavement, chronic illness, business losses, and marital conflict). (American Psychiatric Association 1980, my emphasis)

*The DSM-IV* offers a revised definition of the type of events that might lead to the development of PTSD:

The essential feature of Post-traumatic Stress Disorder is the development of characteristic symptoms following exposure to an extreme traumatic stressor involving direct personal experience of an event that involves actual or threatened death or serious injury, or other threat to one’s physical integrity; or witnessing an event that involves death, injury, or a threat to another person; or learning about unexpected or violent death, serious harm, or threat of death or injury experienced by a family member or other close associate. (American Psychiatric Association 1994)

This revision, in removing the criteria that the event resulting in PTSD must lie outside of ‘usual human experience’, enabled the formal recognition of postpartum PTSD, and this has subsequently been the subject of significant research. There is now widespread recognition that a significant number of women develop PTSD or exhibit symptoms associated with PTSD following childbirth.^12^ A number of studies to date have sought to identify the risk factors and predictors for postnatal PTSD. One recent study suggests, “Traumatic delivery is associated with painful delivery, emergency obstetric procedures, and inadequate care during labor” (Feijo de Mello *et al* 2020, 146). A 2003 analysis suggests “pain experienced during the birth, levels of social support, self-efficacy, internal locus of control, trait anxiety,^13^ and coping were significant predictors of PTSD symptoms after birth” (Soet, Brack, and DiIorio 2003, 36). It continues:

Several characteristics of the childbirth event have been suggested as possible predictors of post-traumatic stress disorder development after childbirth. Two primary characteristics are pain during childbirth and delivery of an ill or stillborn infant. Other environmental factors include hostile and uncaring treatment by medical personnel, the patient’s feelings of powerlessness, inadequate information given to the patient, the patient’s lack of consent, and increasing medical intervention. (37)

Ford and Ayers (2012), drawing on several earlier studies, identify three types of risk factors: ‘the individual vulnerability that women may have before giving birth’, ‘the events of the birth itself and women’s reactions and resources during birth’ and ‘the environment women are in after the birth’ (156). They note that “a history of psychiatric problems and previous trauma is associated with higher risk of having PTSD after birth” (Ford and Ayers 2012), and that “women have a higher risk of PTSD if they have an emergency caesarean or assisted delivery” (157). In addition, they suggest that “women who feel lack of control during birth or who have poor care and support are more at risk of developing PTSD” (157). Dekel *et al* (2017) review of 36 studies on postnatal PTSD concludes that “negative subjective experience of childbirth emerged as the most important predictor” (1). They identify five categories of risk factors: “negative perception of childbirth, maternal mental health, trauma history […], delivery mode and complications and low social support” (5). Under ‘delivery mode and complications’, they list a range of possible risk factors, which include ‘emergency caesarean section’, ‘complications with pregnancy and/or baby’, ‘instrumental delivery’, ‘long labour duration’ and ‘preterm birth’ (7). Significantly, this analysis focuses on studies which looked at ‘full-term successful births’ (2): evidence suggests that the risk of developing PTSD increases in cases where the infant did not survive, as noted by Soet *et al*, who point to stillbirth as a significant risk factor (Soet *et al* 2003, 37).

Dekel, Stueber and Dishy suggest that “Childbirth accounts may be a useful tool to identify at-risk women in the immediate peripartum period” (8). To this end, it is a relatively straightforward procedure to map these various risk factors onto 19th-century case notes of difficult parturitions. However, caution must be exercised in applying these risk factors onto the experiences of women two centuries ago. In particular, ‘negative subjective experience of childbirth’ may be influenced by different expectations, including an awareness of higher maternal and infant mortality rates in childbirth. A greater familiarity with early mortality and stronger religious beliefs may have influenced these risk factors.^14^ Another potentially significant factor may be the lack of available pain relief for much of the 19th century,^15^ leading women to experience different expectations around the pain of childbirth and the manner in which it might be managed. The concept of ‘pain’ is in itself complex, and understanding the historical experience of pain—whether emotional or physical, in childbirth or as a consequence of grief—offers particular challenges (see Bourke 2014). While the pain of childbirth then and now may be comparable, we can speculate that women’s shifting expectations around this may have impacted the manner in which it was experienced. Bourke notes that discourses around pain in childbirth promoted the idea of women’s ‘stoicism’ (207), while religion also played a role here, with some Christian commentators promoting the belief that women’s suffering in childbearing was a consequence of original sin (284). These narratives may well have impacted both attitudes towards and perceptions of pain during labour and delivery. Medical treatment and women’s lack of power or control during childbirth is also likely to have been experienced somewhat differently: in modern society, women are encouraged to consider their birth options beforehand, and there is an expectation that they will be consulted with regard to any interventions carried out during childbirth. In the 19th century, the very different relationship between medical practitioners and birthing women may also have influenced potential risk factors. This article therefore details the potential for women at this time to experience postnatal PTSD based on a range of risk factors, without concluding with any certainty that they necessarily went on to experience symptoms, although the closing section highlights examples of cases of puerperal insanity from 19th-century records in which there appears to be some evidence of traumatic birth as a contributing factor.

More broadly, the wider problems involved in retrospectively diagnosing PTSD in relation to historical events should be considered. In his 1995 work, Allan Young argues that PTSD “is not timeless, nor does it possess an intrinsic unity. Rather, it is glued together by the practices, technologies, and narratives with which it is diagnosed, studied, treated, and represented” (Young 1995, 5). Derek Summerfield argues that “Post-traumatic stress disorder is an entity constructed as much from sociopolitical ideas as from psychiatric ones”, and suggests that “The increase in the diagnosis of post-traumatic stress disorder in society is linked to changes in the relation between individual ‘personhood’ and modern life” (Summerfield 2001). Michaels (2018), in her study on birth and trauma between the 1940s and 1980s notes that “Locating in the historical record what we identify today as trauma in birth is complex and problematic” (63), but nonetheless highlights the “striking consistency over time in how women describe their experiences of traumatic birth” (52). This article, then, while recognising the significant gap between 19th-century birth narratives and modern understandings of postpartum PTSD, argues that the circumstances in which many women gave birth in the 19th century gave rise to some of the most prominent risk factors associated with postpartum PTSD, and that despite the influence of important historical differences, such risk factors were highly likely in some cases to have impacted on women’s postbirth mental health.

In the sections that follow, I examine three specific risk factors of postnatal PTSD (as identified by Soet *et al* and others) in relation to women’s experiences of childbirth in 19th-century Britain: the lack of information available to women and, related to that, their relative powerlessness in the birthing room; medical interventions during childbirth and infant loss. These factors are by no means necessarily distinct from one another: medical interventions in the form of craniotomies, for example, always took place in the context of infant loss—whether before or as a consequence of the intervention. Likewise, it is possible that the more significant the intervention, the greater the sense of powerlessness and lack of control women might potentially experience. Interventions—particularly instrumental interventions—are also associated with greater pain in childbirth, and higher levels of pain would have been experienced by women prior to the regular use of anaesthesia in childbirth. In some cases, instrumental interventions resulted in significant, long-lasting and painful physical trauma. These cases include a combination of risk factors now associated with symptoms of postnatal PTSD, thus potentially increasing their individual risk of deterioration in mental health following childbirth.

## Knowledge, agency, consent

Women’s understanding of the process of childbirth in the 19th century undoubtedly varied considerably from woman to woman and was dependent on a number of factors. Women who had previously given birth would of course have a greater understanding of childbirth than first-time mothers. For women who had not previously experienced childbirth, an awareness of what was involved—and in particular of specific procedures which might be undertaken—was, in some cases, extremely limited. Evidence of this ignorance is to be found in women’s life writing and in medical case notes and literature. Emma Griffin’s work on Victorian working-class autobiographies reveals the extent of this ignorance among some working-class women:

[F]emale writers indicated that knowledge about birth was not passed from mother to daughter. One woman went into labour without knowing ‘where she [the baby] was coming from’. She had thought ‘they cut you up the front’. And this was despite the fact that her mother had seven children and also worked as the local midwife. […] Nor was she alone in her ignorance. Another entered her first labour assuming her baby would ‘come out from the navel’. (Griffin 2020, 100)

Similar accounts are found in *Maternity: Letters From Working Women* Llewellyn Davies (1915). Written in response to a request from Margaret Llewellyn Davies, General Secretary of the Women’s Co-operative Guild, in 1914, these letters detail working-class women’s experiences of motherhood in the late 19th and early 20th centuries. They served one of the (ultimately successful) political aims of the Guild: to exert public pressure on parliament to legislate to improve maternity support and care for all women through the introduction of a maternity and child welfare scheme comprising ante-natal, natal and postnatal support for mothers. The letters thus occupy a unique space between the public and the private, and offer a crucial insight into the maternal lives of working-class women at this time, including their experiences of pregnancy and childbirth, as well as their prior knowledge and expectations. One of the contributors writes:

I had a stepmother who had had no children of her own, so I was not able to get any knowledge from her; and even if she had known anything I don’t suppose she would have dreamt of telling me about these things which were supposed to exist, but must not be talked about. About a month before the baby was born I remember asking my aunt where the baby would come from. She was astounded, and did not make me much wiser. (Llewellyn Davies (1915), 30)

Another contributor details a similar experience:

when I had to have my first baby, I knew absolutely nothing, not even how they were born. I had many a time thought how cruel (not willfully, perhaps) my mother was not to tell me all about the subject when I left home. […] When my baby was born I had been in my labour for thirty-six hours, and did not know what was the matter with me (50).

Middle-class women typically had easier access to the increasing number of advice manuals aimed at wives and mothers, but here too details on birth itself are frequently limited, and the specifics obscured.^16^ Henry Pye Chavasse’s *The Young Wife’s and Mother’s Book* (1842)^17^—one of the most popular of the numerous advice books available for women offering guidance on marriage and motherhood, running to multiple editions throughout the century—is fairly typical in this respect, offering a wealth of advice on pregnancy and infant care, but lacking any detailed information on birth itself. Chavasse refers to ‘the comparative safety of confinements’ and suggests birthing mothers should be assured “that, in the generality of instances, [birth] is a natural process, and that all she has to do is to keep up her spirits, and to adhere strictly to the rules of her medical adviser, and she will do well” (Chavasse 1842, 14).^18^ While such reassurances may have been helpful in the case of straightforward births, they may have had a negative impact on women experiencing traumatic births by keeping women in ignorance of the potential complications of childbirth. Similarly, the anonymously authored *The Mother’s Thorough Resource Book* (1860), which also enjoyed a wide circulation, simply advises the mother to obey ‘the injunctions of the medical man’ (Anon 1860, 20). The section entitled ‘Delivery’ begins: “When the delivery is effected, the patient may place herself composedly upon her back” (Anon 1860). As with other examples of advice literature for expectant mothers, there is no reference to possible interventions by the medical man beyond the instruction to the woman to obey his injunctions. Such evidence suggests that at least some women giving birth for the first time in 19th-century Britain did so with limited or no knowledge of the process of childbirth.

While many women appear to have remained ignorant of the childbirth process, up to and even during delivery, there was some awareness of both the pain associated with childbirth and its potential risks. As Hilary Marland notes on her work on puerperal insanity in Victorian Britain:

Women had long expressed extreme anxiety about their passage through pregnancy and birth, of leaving their infants motherless, or that they would suffer unbearable pain or injury, while the potential death of a child during pregnancy and birth or shortly afterwards was ever present. (Marland 2012, 79)

This is supported by the author of *The Mother’s Thorough Resource Book*, who notes that during pregnancy, “women are apt to become a prey to inquietude and restlessness of mind. The approaching confinement afflicts them with an indescribable terror, and they experience a heavy foreboding and deep despondency, as though some calamity were about to happen” (1860, 12). Modern understandings of the risks associated with developing symptoms of postnatal PTSD suggest that this combination of ignorance and fear may have contributed to the risk of women in the 19th century experiencing birth as traumatic. Indeed, it seems plausible that women entering labour with only a very limited understanding of childbirth would experience feelings of powerlessness, which in turn would potentially impact on their postnatal mental health.

Within the birthing room itself, there is some evidence that women were at times kept in ignorance of the procedures that were being carried out, potentially increasing feelings of powerlessness. In addition to this, the question of consent in the 19th century in relation to interventions in childbirth is by no means clear cut, with medical records and literature suggesting doctors adopted varied approaches to this issue. In an article that appeared in the *British and Foreign Medical Review* in 1845, for example, the question of the consent of the birthing mother in relation to medical interventions in childbirth was debated, with one doctor suggesting that ‘the operation of turning’ (the process of trying to manipulate the baby into a position advantageous to delivery) should be performed ‘without the consent of the patient’, and an attempt made to ‘carefully conceal from her the true nature of [the procedure]’. Another work, by contrast, argues that “the necessity of interference should be impressed on the patient’s mind” and details of the intervention “frankly communicated to her” (Anon 1845, 419).

Robert Lee supports the notion that the patient should be informed of interventions, if possible. In *Lectures on the Theory and Practice of Midwifery* (1844), discussing forceps delivery, he writes:

[I]t is right, before proceeding to apply the blades, to state to the husband and relations, and even to the patient herself, if she is in a condition to comprehend, the reason why you have resolved to trust no longer to the efforts of nature, and even to explain to her what you are going to do. (Lee 1844, 307)

However, there is no indication here that Lee supported the notion of seeking patient *consent* for interventions, and indeed the above statement makes clear that Lee did not always believe it was practicable or advisable even to inform patients of procedures. Debates around patient consent did not begin in earnest until the 20th century, and the general view of the medical profession in the 19th century was that doctors should be trusted to make decisions in the best interests of patients, without necessarily revealing details of those decisions or seeking consent.^19^ Interventions in childbirth—turning, forceps deliveries, destructive operations—were not uncommon in 19th-century Britain, and carried an increased risk of both infant and maternal mortality—not necessarily as a direct result of the intervention (although the use of instruments carried with it a risk of infection), as these were typically employed in cases of difficult deliveries, which themselves inevitably carried a higher risk.^20^ Evidence such as this must lead us to conclude that significant numbers of women underwent some form of intervention (including destructive operations resulting in the death of the infant) without either consenting to or understanding the procedures, all of which carried a risk of infection, as well as physical and/or emotional trauma.

By contrast, there is some evidence to suggest that women exercised more control over decisions on whether or not to induce premature labour. In cases where women suffered from skeletal distortion (due to conditions such as rickets), doctors often advised premature delivery. While prematurity was a key cause of infant mortality,^21^ early delivery offered the possibility of an easier birth, without the need for protracted instrumental deliveries, which often resulted in the death of the baby, and in some cases the mother as well. In the case of Mary Stephenson at Newcastle Lying-in Hospital, doctors advised early induction in subsequent pregnancies following the destructive operation performed on her third child, but their notes from her fourth delivery record that “[t]his advice was not complied with” (Medical Report Book for Newcastle Lying-in Hospital 1821–1833 n.d). Lee also cites a case terminating in a craniotomy in which the patient “had been advised to have premature labour induced but she would not consent, and hoped to get rid of the difficulty by changing her surgeon” (Lee 1849, 39). In another case detailed by Lee, also concluding with the destruction of the child, the mother “would not consent to the induction of premature labour” (Lee 1849, 70). These cases raise interesting questions: a clear hierarchy of power existed between medical men and their (female) patients, with women generally expected to defer to doctors’ superior knowledge and authority, so these refusals in some respects mark a challenge to that authority. It is significant that these refusals take place outside the birthing chamber: control over what happened during labour and delivery was much more difficult, with women not necessarily informed of what was taking place in terms of interventions. The decision by these women to refuse consent to premature delivery of their infants indicates an awareness of high mortality rates among babies born before term. While medical knowledge was largely inaccessible to most women in 19th-century Britain, the risks of premature birth would have been familiar to many—in part through advice literature, which urged women to avoid anything which might result in premature labour or miscarriage (Chavasse 1842, 103).

A case detailed in *The Lancet* provides a tragic example of the multiple traumatic experiences of some women in 19th-century Britain, through the maternal history of a woman aged 34 years, ‘Mrs F’. Over the course of 9 years of marriage, she suffered multiple losses: her first pregnancy ended with the death of the child a few minutes after birth; her second child was stillborn; her third child died at 6 years of age; her fourth pregnancy ended in miscarriage at 5 months; another stillbirth followed; the sixth child was born at 6 months and lived only 12 hours. Her doctor “then recommended the induction of premature labour in the event of another pregnancy. She, however, did not consent” (Forester Wells 1844, 105). Subsequently, her seventh child was delivered by craniotomy. During her eighth pregnancy, she consented to have labour induced at 8 months, and was successfully delivered of a living child. Again, the reason behind this refusal to consent is not made clear, but it might be speculated that there was a desire to avoid this due to the potential dangers it posed to the infant. This raises questions about the extent to which other risks—in particular the risk of the labour terminating in craniotomy—were outlined to patients.

The evidence here is somewhat ambivalent, but it suggests that women were not always provided with the opportunity to give informed consent to the various procedures carried out during labour and childbirth and were, to some extent at least, subject to the authority of both medical men and their husbands. Furthermore, an article published in the *British and Foreign Medico-chirurgical Review* in 1849 points to a lack of sympathy and understanding on the part of doctors towards women refusing to consent to their recommendations:

[W]e must confess that we think in the cases of individuals who, aware of their unfortunate conformation [deformation of the pelvis], allow themselves to become pregnant, and yet refuse their offspring the chance of life which the induction of premature labour gives, the obstetrician is not morally justified in preferring embryotomy to the Caesarean operation. (Capuron 1849, 561)

The implication here is that women who refuse to consent to premature labour should be subject to caesarean sections—an operation with a very high maternal mortality rate in the 19th century, and avoided by many obstetricians for that reason. The suggestion that women ‘allow themselves to become pregnant’ is also troubling, given that married women typically had little to no control over whether or not they became pregnant. Women were encouraged in various ways in 19th-century Britain to submit to the authority of both doctors and husbands, and so their expectations of childbirth may have differed significantly from those of women in Britain today who, typically, exercise significantly more agency over decisions taken in the birthing room. Nonetheless, the powerlessness and lack of control over pregnancy and childbirth that many 19th-century women undoubtedly experienced may have contributed to an increased risk of them experiencing childbirth as traumatic.

## Interventions in childbirth

Feelings of a lack of control and a limited understanding of childbirth may have been exacerbated by the medical interventions employed to assist in delivery in 19th-century Britain. Such interventions may also have resulted in increased pain for birthing women, particularly prior to the regular use of effective pain relief. The risk of complications resulting in maternal and/or infant mortality also tended to increase in cases where medical interventions were required. Infant mortality was obviously inevitable in cases resulting in destructive operations, while other interventions also carried increased mortality risk. Particularly prior to developments in medical understanding around the spread of puerperal fever, which could be introduced via unclean instruments or hands, interventions also posed an increased risk to maternal health. These factors appear to align with recent assessments of the risk factors associated with the development of postnatal PTSD, which Zimmerman (2013) identifies as follows: “unexpected medical interventions, pain beyond the coping ability of the woman, care from providers that was uncaring, unsafe, and inhumane, and the possibility of injury or death for herself or infant”.

Crucially, 19th-century advice literature aimed at mothers-to-be makes little reference to possible interventions during childbirth, and typically simply urges women to follow their medical practitioner’s advice, as reflected in both *The Young Wife’s and Mother’s Book* and *The Mother’s Thorough Resource Book*. This suggests that women were potentially unaware of the possible interventions that might be employed during childbirth prior to labour. One of the women whose experience—and ignorance of—childbirth is detailed in *Maternity: Letters from Working Women* notes that “Instruments had to be used, and I heard the doctor say he could not tell whether my life could be saved or not, for he said there is not room here for a bird to pass. All the time I thought this was the way all babies were born” (Llewellyn Davies 1915, 31). This suggests that a lack of information about the process of childbirth in this case led to an assumption that it was inevitably a highly traumatic experience. In cases of difficult labour, various interventions were commonly employed by medical practitioners in the 19th century. These included ‘turning’: external attempts might be made, but it was often deemed necessary to introduce “the whole hand within the cavity of the womb” (Leishman 1873, 495). Forceps were also used with some frequency to assist in deliveries. In 1819, heir to the throne, Princess Charlotte died shortly after giving birth to a stillborn son. The doctor attending her, Sir Richard Croft, had declined to employ the forceps, and was subsequently criticised for this decision. The publicity surrounding this case may have contributed to the increasing use of forceps in childbirth in the decades that followed. Midwifery literature highlights debates over their use—in particular, relating to when they should be employed, with some practitioners, including Robert Lee, advocating for their use only when the baby’s ear could be felt (1849, 9)—and some case studies from the 19th century indicate long-term physical trauma resulting from the use of forceps.^22^ When delivery could not be completed through either turning or the use of forceps, more extreme instrumental interventions might be employed. As Lee’s work indicates, practitioners in Britain resorted to destructive operations to remove the baby: his published case studies include details of hundreds of births terminating in craniotomies. Caesarean sections were rarely performed, and Lee is representative of other obstetric practitioners at this time in advocating for the preservation of the life of the mother, if necessary at the expense of the child. Consequently, destructive operations which the infant could not survive but which gave the mother a better chance of recovery were generally preferred.^23^

These various interventions could be lengthy and painful, and in some cases resulted in long-term damage and health problems—which might then be exacerbated by further pregnancies and deliveries.^24^ Suturing in cases of perineal trauma became commonplace by the end of the 19th century, but in the decades preceding this, treatment for birth trauma was often limited (see Kettle and Ismail 2016). Consequently, instrumental interventions align with some of the risk factors recently identified in relation to the development of symptoms of postnatal PTSD. A number of Lee’s case studies clearly illustrate this point, citing examples of injuries caused by the injudicious use of instruments by other practitioners, as in his description of a consultation with a woman 9 weeks postpartum, whose child was delivered using forceps:

The perineum, recto-vaginal septum, for about an inch and a half, and sphincter ani, were all destroyed, and the power of retaining the contents of the rectum entirely lost. The case admitted of no relief. This wretched state had resulted from laceration and sloughing of the parts, from the employment of the forceps in her first labour, and immense force exerted to extract the head. The child was dead. (Lee 1849, 19)

In another instance, Lee attended a woman a few days after she had given birth, and describes the injuries caused by the use of instruments, and the apparent injudicious use of forceps by another practitioner:

I was informed that the forceps had been used during the labour by a very young and inexperienced practitioner, and that great extracting force had been employed by him. The symptoms arose, I found, from extensive laceration of the perineum and vagina. It was stated that the labour had not commenced upwards of two hours, when the medical attendant informed […] went home and brought the forceps, which he applied, and after ‘dragging away for a long time with great force, and the head would not come’, he removed the instrument and left the case to nature. The child was born alive 3 or 4 hours after, without assistance. (Lee (1849), 31–2)

In cases in which distortion of the pelvis rendered delivery difficult, craniotomy was often resorted to as the only option likely to preserve the life of the mother, and Lee cites a number of cases of women who underwent this procedure multiple times. Like forceps deliveries, these destructive operations could be lengthy, violent and painful—and offered no hope of the baby’s survival. Lee details several cases of craniotomies lasting a number of hours. In one case, he notes that “The whole head was literally torn to pieces before it could be delivered, and great difficulty was afterwards experienced in drawing the trunk and extremities through the pelvis of the mother” (Lee 1849, 65). In another case, a woman by the name of Mrs Crowther underwent nine deliveries, at least three of which ended in stillbirths. She was delivered by Lee in 1830, at the age of 45, and a craniotomy was performed. The procedure took over 2 hours and resulted in internal injuries leading to incontinence. Though Lee’s case studies do not typically include references to his patients’ personal lives, here he notes that Mrs Crowther’s husband subsequently deserted her, leading to “a life of great indigence and misery” (Lee 1849, 45). He also details another case in which a woman was left with significant internal injuries following a difficult delivery:

The blades of the forceps were […] introduced with great difficulty, and still greater was experienced in getting them to lock. Strong traction was then made for several minutes, and the blades slipped off the head. They were re-introduced, and the efforts to extract renewed and continued till the instrument again slipped off. This happened several times, but the attempt to deliver with the long forceps was not abandoned till the operator was exhausted with fatigue.

Here, too, the woman was rendered incontinent as a result of her injuries, and was also abandoned by her husband, and “reduced […] to the greatest possible misery” (Lee 1849, 5–6). The implication in both these cases is that the marriage breakdowns were linked to the consequences resulting from birth injuries, pointing to the physical and emotional trauma caused by difficult childbirths.

Further examples from Lee’s work also highlight the risks of instrumental deliveries, as in one case from 1851, which again resulted in significant physical injury:

[T]he labour was very protracted and the child was born alive, and the forceps employed frequently, and great force used in extracting the head. [The patient] has never been able to retain her urine since, and has had little or not control over the action of the bowels […] The perineum has been torn into the rectum; cannot now retain the contents of the rectum thoroughly (Lee 1864, 53–4).

While Lee’s work offers the perspective of the medical man, several cases of instrumental delivery resulting in injury are also found in *Maternity: Letters From Working Women*, which offers us a glimpse into birthing mothers’ experiences of difficult deliveries. One woman details her experience of a lengthy and difficult labour: “after all that suffering, [I] had to be delivered by instruments, and was ruptured too badly to have anything done to help me. I am still suffering from the ill-effects to-day. This is thirty-one years ago. […] I was unable to sit down for 3 months” (Llewellyn Davies 1915, 70). Another, delivered via instruments while under the influence of chloroform recalls, “I was a cripple for nearly twelve months” (72), suggesting extensive birth injuries. One correspondent who underwent an instrumental delivery wrote: “The doctor said that my baby could not have been born without [intervention]. No doubt it relieved me at the time, but I suffered afterwards, and I was all torn with the instruments, and had to be stitched” (142). Another recalls the consequences following the birth of her fifth child: “I was so injured that for nearly ten years I was an invalid” (122). While correspondents typically reference the physical consequences of instrumental deliveries, one woman’s words suggest the psychological impact following a difficult instrumental birth. She writes that the ‘forced birth’ (presumably forceps delivery) of her child “is too terrible to go through even now after twenty-eight years. Suffice it to say that next morning there was a poor little baby boy with a very large swollen head dreadfully cut, and a young mother dreadfully cut also” (119).

The collection also includes two letters from women who underwent embryotomies—rare, if brief, accounts of this procedure from the mothers’ perspectives. The first writes: “My last baby was literally torn from me. The doctor told my husband he could not save both. They dare not chloroform me, and so I had to bear it” (Llewellyn Davies 1915, 116). In the second case, the woman appears to have undergone the procedure five times due to ‘deformed pelvis bones’, though she also successfully delivered five live children: “I have five fine healthy girls, but the boys have all had to have the skull-bones taken away to get them past the pelvis. Always a case for two or three doctors, so you will know I have suffered something” (167). She notes that she was unable to walk for 11 months after the birth of her first child. There can be little doubt that such extensive physical trauma must also have had an emotional and psychological impact.

## Infant loss

As evidenced above, instrumental births posed a threat to both infant and maternal health, and in the case of craniotomies and embryotomies, inevitably resulted in the loss of the child. The death of an infant in childbirth is now clearly identified as a risk factor for postnatal PTSD, as Soet *et al* and others have confirmed. Again, caution should be urged here in applying this wholesale to the experiences of women in 19th-century Britain.^25^ Infant loss—either in childbirth or in early childhood—was a reasonably common occurrence: in some areas, infant mortality was as high as 25% (see Woods and Shelton 1997, 48).^26^ These figures do not include stillbirths (including those lost as a result of destructive operations), which were not formally recorded until 1926.^27^ It is also worth stressing that—particularly in the context of a widespread lack of control over reproduction and fertility—we cannot assume that infant loss was necessarily experienced as traumatic. Nonetheless, assuming maternal grief was experienced with less intensity due to its increased occurrence—particularly among the poor—is potentially problematic, as Julie-Marie Strange makes clear in her study on death, grief and poverty in Victorian Britain, despite the moral panic around the issue of infanticide (Strange 2005, 230–62).

Although complications potentially resulting in the death of the baby could arise in any delivery, some women, as a consequence of their own health problems, were more liable to experience this loss than others. This was particularly the case for women suffering from distortion of the pelvis—often caused by rickets, and thus partly linked to poverty (poor diet, lack of sunlight). This condition, evidenced by the hundreds of cases detailed by Lee, frequently resulted in birth via craniotomy. Consequently, some women—such as Mary Stephenson at the Newcastle Lying-in Hospital—suffered multiple losses during repeated deliveries. A letter published in *The Lancet* in 1847 alludes to one woman with a very small pelvis who delivered six stillborn children—some via craniotomy. Premature induction of labour—one of the only alternatives to fetal destruction in women suffering from distorted pelvises, but itself linked to high infant mortality—led to the birth of a live child, who lived 3 years (Wordsworth Poole 1874, 11). Lee details several cases of women who underwent multiple destructive operations to enable delivery. He describes the case of one woman with a small pelvis, whom he attended in 1844 during her fourth labour:

Her first [labour] had been tedious and difficult, and the child was born dead, without artificial assistance. The second child was born dead in the sixth month. In the third pregnancy, she went to the eighth month; the feet presented; the labour was long and difficult, and the child was extracted dead. (Lee 1849, 66)

Her fourth birth ended in a similar manner: after a long and difficult labour, the child appeared to have died, and Lee performed a craniotomy in order to extract the body. In another example included in *Three Hundred Consultations in Midwifery*, Lee describes the experience of a woman suffering from a distorted pelvis delivering her fifth child—her first died soon after birth and the remainder were stillborn, with two delivered by craniotomy. On this occasion, she was attended by four practitioners and subject to lengthy and invasive interventions, including turning and ultimately a craniotomy. Following the birth of this fifth child, the patient showed signs of a ruptured uterus and was not expected to survive (Lee 1864, 50).

*Maternity: Letters from Working Women* also details the experiences of women who have suffered miscarriages, stillbirths and infant loss—in some cases multiple times. In a short section at the end of the volume, it provides statistics related to 348 cases for which details were received in the preparation of the volume. These show a miscarriage rate of 15.6 per 100 live births and a stillbirth rate of 5.9 per 100 live births (Llewellyn Davies 1915, 194).^28^42.4% of the mothers had suffered stillbirth, miscarriage or both. 41 mothers suffered multiple miscarriages, and 14 multiple stillbirths. In 8.7% of live births, the child died before it reached 12 months, and “50 per cent of the deaths occurred within the first month or from ante-natal or natal causes after the first month” (195). A total of 24.7% of the mothers “lost children in the first year of life” (195). While the work of the Women’s Cooperative Guild gave voice to the experiences of working-class women, wealth and social status were no guarantee of protection from traumatic maternal experiences.^29^

Expectations related to life expectancy and awareness of the risks associated with birth and the first 12 months of life in particular—as well as other potentially relevant factors, such as religious beliefs—mean more recent work which identifies infant loss as a risk factor for postnatal PTSD cannot be said to be directly applicable to the 19th-century context. Nonetheless, as recent definitions indicate, PTSD is associated with events ‘outside the range of usual human experience’, therefore the loss of an infant, even within the context of the 19th century when such losses were relatively frequent, may still have increased the risk of the onset of symptoms today associated with postnatal PTSD.

## Conclusions

The multiple examples cited above demonstrate that over the course of the 19th century, many thousands of women experienced difficulties in childbirth that are now identified as risk factors for postnatal PTSD. While some of these cases reference long-term physical damage, few make any reference to the psychological or emotional impact of difficult births and infant loss—though we might reasonably infer this from, for example, references to women’s husbands abandoning them following difficult births, and the long-term physical suffering mentioned in some cases. However, if 19th-century medicine did not explicitly recognise postpartum PTSD as a disorder, there is evidence of some understanding of the relationship between traumatic births and mental illness in discourses around puerperal insanity.^30^ While some works on the subject of puerperal insanity were circumspect with regard to this link—pointing to a vaguer association between ‘bodily weakness’ (Brudenell Carter 1855, 136–7) and the onset of mental disorder, for example—others point to traumatic births as a key cause of puerperal insanity. One work from 1818, for example, cites ‘difficult parturition’ as one of the key causes of insanity in women (Saunders Hallaran 1818, 50). Another, from 1859, suggests that women are particularly vulnerable to mental deterioration during the period “soon after delivery when the body is sustaining the effects of labour” (Gooch 1859, 54). Several case studies seem to support this connection. One case cited in an article on puerperal insanity published in the *Medical Times* in 1874, for example, describes a woman aged 23 years who, during an instrumental delivery, “became violently excited, and charged her husband with attempts to murder her by means of the forceps” and then “Tried to choke the physician”. She subsequently continued ‘deranged and under restraint about 2 weeks’ (Anon 1874, 396–7). The article goes on to refer to the “many cases [of puerperal insanity] which arise during instrumental deliveries” (397). While the purpose of this article is not to retrospectively diagnose cases of postnatal PTSD in 19th-century women, nonetheless, such cases are revealing in terms of the manner in which many women experienced childbirth in 19th-century Britain.

Several factors, including increased infant and maternal mortality, the use of destructive operations, a lack of available or effective pain relief and the increased likelihood of previous difficult births meant that women in 19th-century Britain were statistically more likely than birthing women today to be exposed to what are now recognised as key risk factors for postpartum PTSD. The extent to which such circumstances existed *as potential risk factors* in the 19th century is, inevitably, difficult to ascertain, but it is worth emphasising that the normalisation and common occurrence of these various circumstances did not necessarily detract from their potential status as risk factors. The revision of *The DSM* definition of PTSD to remove the qualification of an experience as ‘outside the range of usual human experiences’ clearly supports the idea that experiences that lie within the everyday or the ordinary may in fact lead to the onset of symptoms of PTSD. It is clear from an examination of case studies of difficult parturitions in 19th-century Britain that women regularly encountered some of the identified risk factors of postpartum PTSD, while some case records and discourses around puerperal insanity from the period clearly support the idea that traumatic birth experiences, then as now, impacted women’s mental health.

## Data Availability

Data sharing not applicable as no datasets generated and/or analysed for this study.

## Footnotes

On childbirth in 19th-century Britain, see Cox (2023); Begiato (2017); Badger (2014); Marl(2004a, b); Marks (1993). On childbirth in the 18th century, see Fox (2022).

There was no legal requirement to record the details of infants born dead—either through stillbirth or as a consequence of craniotomies performed during labour—so accurate estimates are impossible. However, it is clear from medical records and midwifery literature that destructive operations were widely performed with some frequency. Robert Lee’s *Three Hundred Consultations in Midwifery* (1864), for example, details almost 100 cases.

The two terms are often used interchangeably in 19th-century sources. Craniotomy—the opening of the infant’s head in order to reduce its size and allow delivery—is a form of embryotomy (surgical destruction of the unborn fetus). Evidence suggests craniotomy was the most commonly used form of the procedure during this period. Estimating the frequency of these operations is challenging, as stillbirths were not formally recorded, but records from lying-in hospitals, such as Newcastle, and midwifery literature such as the works of Robert Lee point to their common use in cases of difficult deliveries.

One source suggests a maternal mortality rate of over 90% (Lee 1849Lee 1849, 11).

In 1823, obstetrician James Blundell proposed tubal ligation as a solution for women with distorted pelvises to ensure avoidance of pregnancies (see Young 1964), but such procedures were not effectively developed until the late 19th century.

The case notes for her third delivery indicate she was prescribed an opiate on the evening of her fourth day in labour. There is no reference to any further pain relief being administered ahead of or during the lengthy procedure to remove the child, which took several hours.

On the use of induction of premature labour in 19th-century obstetrics, see Playfair (1865).

Marital rape was not criminalised in the UK until 1991.

Typically, 19th-century medical case notes only evince an interest in these issues if there is a clinical concern—if the emotional state pertains to a physical complaint or diagnoses of puerperal mania, for example. Robert Lee’s work is representative of this trend. For recent work on maternal emotion among the Victorian working classes, see Griffin (2018).

Robert Lee was a Scottish obstetrician, and for over 30 years Chair of Midwifery at St. George’s Hospital, London. He published extensively on midwifery and obstetrics, his most notable works being *Clinical Midwifery* (1842), *Lectures on the Theory and Practice of Midwifery* (1844) and *Three Hundred Consultations in Midwifery* (1864). See Marl(2004a, b).

I refer primarily to Lee’s *Clinical Midwifery* (second edition, 1849, ‘comprising the histories of five hundred and forty-five cases of difficult, preternatural and complicated labour’) and *Three Hundred Consultations in Midwifery* (1864). The former details cases dating from the 1820s to the 1840s, and the latter cases dating from the 1840s to the 1860s.

A meta-analysis by Soet *et al* (2003) suggests the percentage of women ‘experiencing birth as traumatic may fall between 20% and 30%, and of developing post-traumatic stress disorder (PTSD), between 2% and 6%’ (37). A 2012 analysis suggests similar figures: ‘the rate of PTSD in women following childbirth is between 1.5% and 5.6%, depending on the time-point at which symptoms are measured’, in addition to which ‘20%–30% of women rate birth as traumatic’ (Ford and Ayers 2012, 156).

‘Trait anxiety’ refers to ‘A person’s general or characteristic level of anxiety’ (see Colman 2015).

Religious beliefs influenced women’s experiences of childbirth in various ways: opposition to pain relief was sometimes couched in religious terms; debates over the use of destructive operations at times employed religious language (Robert Lee was accused of ‘outheroding Herod’ due to his use of such procedures—see Anon 1842, 130) and indeed the use of these procedures was severely limited in Catholic countries where the life of the infant was deemed sacrosanct. There was also some debate over the issue of in-uterine baptism in cases where the infant was not expected to survive the birth: Ryan (1841), for instance, in A *Manual of Midwifery*, which ran to multiple editions in the mid-19th century, suggests there is not “any scriptural authority […] that alludes to intra-uterine baptism” (246).

Chloroform was first used in childbirth in the 1840s. Use of pain relief throughout the period partly depended on what medical attendance could be afforded.

On 19th-century advice literature for mothers and its echoes in contemporary culture, see Åström (2015).

Chavasse published a number of popular works on motherhood. They ran to numerous editions in both Britain and America, and also appeared in multiple translations.

Later editions of Chavasse’s work do provide more detail on labour and childbirth, reflecting a wider trend in the late 19th century.

This attitude is reflected in Thomas Percival’s *Medical Ethics* (1803), which remained influential throughout the period.

On maternal and fetal mortality rates in cases of forceps deliveries, see Galabin (1877).

The *Annual Report of the Registrar General* throughout the period cites premature birth as a leading cause of death. As an example, in 1855, premature birth was cited as responsible for almost 18 000 deaths in England and Wales, and as the sixth leading cause of death that year (Farr 1857, 187).

There was significant debate among obstetricians throughout the 19th century on the use of forceps: when they should be employed, and what type should be used (see McClintock 1876). Lee in his published works is frequently critical of other practitioners’ injudicious use of forceps (see, eg, Lee 1849, 44).

On the continent, caesareans were performed somewhat more frequently due to the influence of Catholic doctrines on attitudes towards those operations which destroyed the infant.

See, for example, case number 71 in Lee’s *Three Hundred Consultations in Midwifery*.

On women’s experiences of pregnancy loss in 19th-century America, see Withycombe (2018).

Woods and Shelton note that these figures may not include all infant deaths, because of the likelihood that “not all live births were registered” (47), so the true figure may have been even higher in some areas.

Consequently, estimating the number of stillbirths in the 19th century is difficult. Nineteenth-century epidemiologist and statistician William Farr estimated a stillbirth rate of approximately 3%. This led him to conclude that in 1852 alone, there were approximately 22 122 stillbirths in England and Wales (see Humphreys 1885, 270).

This is considerably higher than that estimated by Farr, potentially reflecting the fact that the volume is concerned with the experiences of poorer women.

Multiple cases evidence this. Adelaide of Saxe-Meiningen, for example—Wife of William IV—suffered at least one miscarriage and gave birth to three stillborn children, including twin boys, as well as two daughters who died—one only a few hours after birth, and the other at 4 months old.

Such understandings of a causal link between difficult births and psychological distress date back much further than the 19th century. See, for example, Philippa Carter’s article on the case notes of early modern physician Richard Napier (2021).
